# The Network of Cytokines in Brain Metastases

**DOI:** 10.3390/cancers13010142

**Published:** 2021-01-05

**Authors:** Jawad Fares, Alex Cordero, Deepak Kanojia, Maciej S. Lesniak

**Affiliations:** Department of Neurological Surgery, Feinberg School of Medicine, Northwestern University, Chicago, IL 60611, USA; jawad.fares@northwestern.edu (J.F.); alex.cordero@northwestern.edu (A.C.); deepak.kanojia@northwestern.edu (D.K.)

**Keywords:** cytokines, chemokines, interferons, interleukins, lymphokines, tumor necrosis factors, brain metastasis, brain tumor

## Abstract

**Simple Summary:**

Cytokines are small proteins that impact health and disease. They regulate cell signaling and have been shown to affect the immune response to various diseases, including cancer. Brain metastasis is a deadly disease. When cancer from the lungs, breast, or skin spreads to the brain, the survival of patients decreases. Therefore, understanding how cytokines affect and modulate the metastatic spread of cancer to the brain can help in improving diagnostic capabilities and therapeutic outcomes.

**Abstract:**

Brain metastases are the most common of all intracranial tumors and a major cause of death in patients with cancer. Cytokines, including chemokines, interferons, interleukins, lymphokines, and tumor necrosis factors are key regulators in the formation of brain metastases. They regulate the infiltration of different cellular subsets into the tumor microenvironment and affect the therapeutic outcomes in patients. Elucidating the cancer cell-cytokine interactions in the setting of brain metastases is crucial for the development of more accurate diagnostics and efficacious therapies. In this review, we focus on cytokines that are found in the tumor microenvironment of brain metastases and elaborate on their trends of expression, regulation, and roles in cellular recruitment and tumorigenesis. We also explore how cytokines can alter the anti-tumor response in the context of brain metastases and discuss ways through which cytokine networks can be manipulated for diagnosis and treatment.

## 1. Introduction

Cytokines and chemokines are soluble signals that control the migration and positioning of cells in a specific microenvironment [1]. They are released by immune cells, endothelial cells, fibroblasts, and other stromal cells, and act by binding to cell surface receptors on effector cells. The immune system is particularly dependent on cytokines and chemokines for coordinated function and response to pathogens, thus favoring the proper conditions for an optimal adaptive immune response [2]. Cytokine release is usually triggered by growth factors, foreign stimuli, and/or other cytokines. In cancer metastases, the release of cytokines and chemokines activate cellular signaling pathways that support the invasion of cancer cells at the primary tumor site, interactions of cells with the extra cellular matrix (ECM), and the successful colonization of cancer cells in secondary organs [3]. Preclinical studies on human cancers and mouse models show that the interaction between cytokines and cancer cells increases metastases [4].

Brain metastases are the most common malignant brain tumors and a major cause of death in patients with cancer. They require the invasion of primary cancer cells from the lungs, breast, or skin, trafficking through the circulatory system, and the colonization of the brain parenchyma [5]. Cytokines and chemokines secreted by brain metastatic cancer cells, stromal cells, immune cells, and other cells within their surrounding microenvironment drive the various stages of metastasis [4]. They mediate the brain response to metastatic cells by directing the trafficking of leukocytes into the tumor microenvironment. These proteins exert their effects either through autocrine or paracrine mechanisms to facilitate the cross-talk between the metastatic cancer cells and their colonized niche. The migration of cells that express a specific chemokine receptor occurs across a chemokine gradient that allows cells to move toward high local concentrations of chemokines. This migratory response is complex and consists of diverse leukocyte subsets with both antitumor and pro-tumorigenic activities [6]. Preclinical reports show that chemokine-receptor antagonists can decrease the infiltration of immune cells of myeloid origin and thus induce the arrest of metastatic growth and spread in the brain [7].

Understanding the molecular underpinnings that govern the cancer cell-chemokine interactions in the setting of brain metastases is crucial for the development of more accurate diagnostics and efficacious therapies. In this review, we focus on cytokines and chemokines that are found in the tumor microenvironment of lung, breast, and melanoma brain metastases; we elaborate on their trends of expression, regulation, and roles in cellular recruitment and tumorigenesis. We also explore how these cytokines alter the anti-tumor response and discuss ways through which chemokine networks can be used for potential treatments.

## 2. Cytokines in Lung Cancer Brain Metastases

Lung cancer frequently spreads to the brain. It is estimated that up to 40% of patients with non-small cell lung cancer (NSCLC) will eventually develop brain metastases at some point during the course of their disease [8]. Of patients with brain metastases, lung cancer is the primary tumor in 40–50% of cases [9,10]. Once brain metastases ensue, the prognosis is poor, with life expectancy usually being under a year. Cytokine and chemokines have been reported to play integral roles in the process of lung cancer brain metastases. They are involved in pre-conditioning the metastatic niche in the brain for cancer growth and survival, interacting with resident cells in the tumor microenvironment of the brain, and mediating the immune response to the metastatic lung cancer cells [3] (Table 1).

Pre-conditioning the brain microenvironment with specific cytokines, chemokines, or tumor–secreted exosomes enhances lung cancer cell outgrowth in the brain. Transforming growth factor-β1 (TGF-β1) is well known for its role in epithelial to mesenchymal transformation (EMT). Pre-treatment of lung cancer cells with TGF-β1 in mouse models leads cells to metastasize almost 3 times more than wild types toward the brain [11]. TGF-β1 genotype rs1982073 is associated with poor brain metastasis-free survival in patients with NSCLC who underwent radiation therapy [25]. Pending further validation, this genotype can serve as a useful predictor of outcomes in this subset of patients. Genotype variants in the TGF-β signaling pathway can also serve as predictive biomarkers of brain metastases. By analyzing DNA from blood samples, the GG genotype of SMAD6 rs12913975 and TT genotype of INHBC rs4760259 were associated with an increased risk of brain metastases in patients with NSCLC [13]. Pre-B-cell leukemia homeobox (Pbx)-regulating protein-1 (PREP1) is a ubiquitous homeoprotein that functions as an EMT inducer and is a pro-metastatic transcription factor. PREP1 accumulation has been detected in brain metastases of various solid tumors, including NSCLC [14]. Further analysis showed that PREP1 promoted metastasis in the brain through controlling the TGF-β-SMAD3 pathway [14]. CC chemokine ligand 2 (CCL2) induces visfatin upregulation [15]. Visfatin is a pro-inflammatory adipocytokine that mediates the transmigration of small-cell lung cancer (SCLC) cells across the blood-brain barrier (BBB) [15].

The colonization of the brain parenchyma by metastatic lung cancer cells involves the release of cytokines and factors that facilitate the communication between the tumor cell and its microenvironment. Tumor necrosis factor-α (TNF-α) enhances the adhesion of CD15, which is expressed at high levels in metastasizing lung cancer cells to the brain, and E-selectin, which is expressed on brain endothelial cells [16]. TNF-α, cystatin C, cathepsin L, insulin-like growth factor-binding protein 7 (IGFBP7), and vascular endothelial growth factor (VEGF) are secreted by NSCLC cells metastasizing to the brain [12]. These factors damage the endothelial glycocalyx, which subsequently leads to upregulation in E-selectin and improved mediated adhesion of metastasizing cells to the brain microvascular endothelium [12]. Even before the formation of brain metastases, the cerebral metabolic status of patients with lung cancer is altered. Glutamate, creatine, and phosphocreatine are significantly lower in the cortex of the patients [26]. The concentration of TNF-α is inversely correlated with the concentration of N-acetyl-aspartate, an indicator of mitochondrial oxidative capacity, in the occipital cortex [26]. Cell migration-inducing and hyaluronan-binding protein (CEMIP) is elevated in exosomes from brain metastatic cells [17]. Uptake of CEMIP+ exosomes by brain endothelial and microglial cells induces inflammation in the perivascular niche by upregulating the pro-inflammatory cytokines encoded by *Ptgs2*, *Tnf*, and *Ccl/Cxcl*, which are known to promote brain vascular remodeling and metastases [17]. Astrocytes in the tumor microenvironment are activated by tumor cell-derived factors, such as the macrophage migration inhibitory factor (MIF), IL-8, and plasminogen activator inhibitor-1 (PAI-1) [18]. Activated astrocytes, in turn, produce IL-6, TNF-α, and IL-1β, which promote tumor cell proliferation [18]. The astrocyte-tumor interaction increases the expression of receptors for IL-6 and its subunit gp130 and decreases the receptors for TNF-α and IL-1β on HARA-B metastatic lung squamous carcinoma cells [18]. Tumor-derived IL-6 is capable of inducing programmed death-ligand 1 (PD-L1) expressing myeloid cells in vitro [19]. The frequency of PD-L1+ myeloid cells correlates with the presence of brain metastases. Patients with brain metastatic lung carcinoma demonstrated increased peripheral monocyte PD-L1, MDSC abundance, and Treg percentage compared to controls [19]. Adding brain-metastasis-conditioned media to lung cancer cells increases monocyte PD-L1; IL-6 levels in conditioned media further correlated with PD-L1 induction [19]. Treatment with anti-IL-6 or anti-IL-6 receptor antibodies reduces PD-L1 expression patient-derived xenografts, which indicates that tumor-induced peripheral immunosuppression promotes brain metastases [19].

Growth factors and cytokines in the tumor microenvironment play a role in the survival of metastatic cancer cells in the brain. Upon rapamycin treatment, IL-1, IL-3, IL-6, TNF-α, TGF-β, PDGF, MCP-1, and MIP-1 expression were higher in murine models of NSCLC brain metastases, but IGF-1 expression was lower compared to controls [27]. Interestingly, colony stimulating factor 1 (CSF-1) can reprogram myeloid cells, specifically into tumor-promoting macrophages in the brain parenchyma [20].

Analyses of immunological markers could potentially serve as prognostic markers in patients with lung cancer brain metastases. IL-2 and IL-7 can serve as independent predictors of survival in patients with brain metastases [21]. IFN-γ responses to mesothelin, a surface-bound antigen that is overexpressed in several malignancies, are conditioned by IL-2 and IL-7 [21]. CD37, cystatin A, and IL-23A are differentially downregulated in patients with lung cancer brain metastases [28]. The validation of these biomarkers could have implications on surveillance patterns in patients with brain metastases from NSCLC [28]. IL-17, released by Th17 helper T cells, is markedly increased in the serum and cerebrospinal fluid (CSF) of patients with lung cancer brain metastases [29]. The IL-6 receptor on tumor cells was upregulated during astrocyte-mediated activation, which suggests that this receptor can be a therapeutic target to inhibit the growth of the metastasized lung tumor cells in the brain [30]. An isogeneic comparison of primary and metastatic lung cancer cells identified that the downregulation of CX3CR1 in lung adenocarcinomas causes more metastatic spread to the brain [31]. Intracranial metastatic tissue samples of lung cancer show significantly higher expression of nitric oxide synthase, cytoskeleton protein caldesmon, and OPN [22]. Nitric oxide can remodel the cytoskeleton and promote the mobility of lung cancer cells [22]. The expression of chemokine CXCL12 and its receptor, CXCR4, is significantly higher in NSCLC samples of patients with brain metastases [32], which allow for the differentiation between NSCLC patients without and with brain metastases, with good diagnostic accuracy and adequate predictive power [32]. Interestingly, the gene expression profiling of metastatic lung adenocarcinoma in the brain shows an increased expression of the receptor-binding cancer antigen expressed on SiSo cells (RCAS) and Fas ligand (FasL), which are present in neoplastic cells, induce apoptosis of NK/T cells, and play a role in immune evasion [23]. In addition, an immunohistochemistry analysis revealed a reduced expression of interleukin 13 receptor alpha2 (IL-13Ralpha2) in brain metastases compared to primary tumor cells [23]. Moreover, Met receptor and its ligand, hepatocyte growth factor (HGF), are commonly overexpressed in NSCLC [24]. HGF/Met co-overexpressing cells demonstrated enhanced tumorigenicity and higher spontaneous metastases to the brain [24].

## 3. Cytokines in Breast Cancer Brain Metastases

Breast cancer is the most frequent cancer among women, impacting 2.1 million women per year globally. It constitutes the greatest number of cancer-related deaths in women and has one of the highest risks for intracranial spread [33,34]. The presence of specific cytokines and chemokines has been associated with the metastatic spread of breast cancer to the brain (Table 2). Cytokines and chemokines can play a role in enhancing transmigration across the blood-brain barrier, promoting immunosuppression in the tumor microenvironment, and facilitating the colonization of metastatic cells in the brain parenchyma [3,4,5].

Cytokines and growth factors can alter the permeability of the blood-brain barrier. CX3CL1 and CXCL13 were found to be elevated in the sera of patients with breast cancer brain metastases [35]. Treatment of the endothelial cells that constitute the BBB with the sera of patients with breast cancer selectively increases the expression of CXCL13 and the permeability across the barrier using fluorescein [35]. GRO-α, ICAM-1, IL-6, IL-8, GM-CSF, and CCL5 also facilitate the transmigration of breast cancer cells across the BBB [36,37,38,39,51]. The silencing of syndecan-1 increased the release of these cytokines by invading cancer cells [40].

Metastatic breast cancer cells in the brain use cytokines to suppress the immune microenvironment and promote tumor survival. Granulocyte colony-stimulating factor (G-CSF) recruits Arg1+ and PD-L1+ immunosuppressive neutrophils into the brain to drive metastasis outgrowth. G-CSF secretion is regulated by the phosphorylation of the enhancer of zeste homolog 2 (EZH2) at tyrosine-696 (pY696), which switches EZH2’s function from a methyltransferase to a transcription factor that increases c-JUN expression. c-Jun upregulates pro-tumorigenic inflammatory G-CSF [41]. G-CSF-blocking antibodies or immune checkpoint blockade therapies combined with Src inhibitors impeded the formation of brain metastases in multiple mouse models [41]. Rapidly progressing brain metastases contained many enlarged blood vessels. The expression of VEGF by breast cancer cells directly correlated with angiogenesis and the growth of brain metastases [42].

C-X-C chemokine receptor 4 (CXCR4) and its ligand stroma-derived factor 1 (SDF1) are upregulated in various cancers, and CXCR4 inhibition prevents metastasis formation [61]. In breast cancer brain metastases, CXCR4 is upregulated in microglia [43], which supports the invasion of breast cancer cells into the brain [62]. Monocyte chemoattractant protein-1 (MCP-1) is also implicated in breast cancer progression in the brain. A high level of MCP-1 in breast cancer cells was shown to promote the migration and infiltration of the macrophage into the brain through its receptor CCR2 [49,50]. GM-CSF has a similar effect as MCP-1 in enhancing microglial proliferation [52]. Breast cancer brain metastases exhibit a high level of expression of CX3CL1 [63,64], which functions as a chemoattractant for macrophages and microglial cells [53]. These microglia/macrophages release cytokines and chemokines, such as IL1-β and TNF-α, that stimulate brain microvessel endothelial cells, leading to an increased permeability of the blood-brain barrier [54,55] and immune cell infiltration from the peripheral system. Osteopontin, through its receptors, CD44 and integrin α(V)β(3), plays a key role in macrophage chemotaxis, a mechanism that may be utilized by metastatic brain tumors in the process of dissemination [65].

Astrocytes also produce SDF1, which upon binding to CXCR4 triggers a downstream signal transduction that induces the production of miR345 [44]. miRNA345 silences the production of KISS1 [45], which can lead to the upregulation of proangiogenic VEGF [46], pro-invasive MMP9 [47] and SLUG [48], EMT-related E-cadherin [45], autophagy-related ATG5, LC3-II, and p62/SQSTM1 [44], to ultimately promote tumor cell adaptation and propagation in the brain. Breast and lung cancer cells express protocadherin 7 (PCDH7), which assembles cancer cell-astrocyte gap junctions that are made up of connexin 43 (Cx43) [56]. Upon channel formation, brain metastatic cancer cells transfer the second messenger cGAMP to astrocytes to activate the STING pathway [56]. This causes the release of inflammatory cytokines such as IFNα and TNF that activate the STAT1 and NF-κB pathways in brain metastatic cells, thereby promoting tumor growth and resistance [56]. Gene expression profiling using cDNA microarrays in breast cancer brain metastases showed that the expression of astrocyte-derived cytokine receptors, such as IL-6 receptor [66], TGF-beta receptor, and IGF receptor, were significantly increased [57], indicating that cytokines produced by glial cells contribute to the metastatic process.

A proteomic analysis of the secretome in breast cancer brain metastases showed that several secreted proteins were differentially altered when compared to patients without brain metastases. The pathway analysis shows that TGF-β1 is a top upstream regulator in all metastatic breast cancer cells. Fibronectin 1, a protein involved in tumor progression [67] and invasion [58], is decreased in metastatic breast cancer cells to the brain as compared to other secondary sites, suggesting a brain-specific phenotype [59]. Insulin-like growth factor-binding protein 7 (IGFBP7), which has several characteristics of a potential tumor suppressor, is also decreased in brain-specific metastases [59].

Immunogenic therapies that use anti-tumorigenic cytokines are being developed in breast cancer brain metastases. A cellular vaccine consisting of allogeneic fibroblasts modified to secrete IL-2 significantly increased survival in animal models. A histopathological examination revealed tumors associated with lymphocytic infiltrations [68].

## 4. Cytokines in Melanoma Brain Metastases

Melanoma has the highest propensity to metastasize to the brain compared to other cancers, resulting in significant morbidity and death [69]. Once disseminated in the brain, melanoma cells communicate with brain resident cells that include astrocytes and microglia. This complex cross-talk between immune cells and brain metastatic melanoma cells induces the production and secretion of cytokines and chemokines (Table 3).

The formation of melanoma metastases in the brain is preceded by early changes in the brain microenvironment that include the breakdown of the BBB, vascular hyperpermeability, and reactive astrogliosis. Studies using a melanoma brain metastasis immunocompetent mouse model revealed an upregulation in proinflammatory cytokines CXCL10, CCL17, CCL2, IL6, and IL-1β [87]. CXCL10 is secreted in response to IFN-γ by various cell types, including astrocytes, fibroblasts, and endothelial cells, and was shown to modulate the migration of monocytes, macrophages, T cells, and natural killer (NK) cells to the brain [88]. Importantly, CXCL10 levels are elevated in advanced melanoma patients, and were associated with poor clinical outcomes [89,90]. In addition, CXCR3, the receptor for CXCL10, is upregulated in brain-tropic melanoma cells [70]. Interestingly, immunokine profiling studies in the cerebrospinal fluid (CSF) of advanced melanoma patients showed that elevated levels of CXCL10, CCL17, and CCL4 may correlate with a more aggressive development of brain metastases [91].

The chemokine motif receptor 4 (CCR4) and its ligands CCL17 and CCL22 are regulators of immune responses, especially those mediated by regulatory T cells (Tregs) and TH2 cells [91,92]. The expression of CCR4 was significantly higher in paired clinical specimens of melanoma metastases than in samples of primary tumors from the same patients [71]. Their results demonstrated that CCL17 (but not CCL22) was sufficient to enhance melanoma cell invasiveness in the brain, and blocking CCR4 in vivo using a CCR4-antagonist small molecule reduced the tumorigenicity and micrometastasis formation of melanoma cells. CCL2, also known as MCP-1, has been reported to bind CCR4 on cytotoxic T lymphocytes, resulting in their recruitment to the metastatic melanoma cells and inducing an immune-mediated protective role [72]. Moreover, the brain microenvironment induces a loss of PTEN expression in metastatic melanoma cells, leading to an increased secretion of CCL2 and a subsequent recruitment of myeloid cells that enhance the outgrowth of brain metastatic melanoma cells via enhanced proliferation and reduced apoptosis [73]. Another study using human melanoma brain metastasis xenografts showed that metastatic melanoma cells stimulated with CCL22 showed a differential AKT phosphorylation pattern [74], which is associated with tumor cell survival and proliferation [93]. This hints at the importance of the CCL22-CCR4 axis in the process of brain metastases in human melanoma.

The chemokine/receptor system CXCL12/CXCR4 plays a key role in multiple biological functions and is one of the most investigated chemokine-receptor axes in the metastatic process. Indeed, CXCR4 expression might be a powerful prognostic marker in malignant melanoma tumor cells [94,95]. In addition, other studies highlight the importance of CXCR7, another CXCL12 receptor expressed mainly in endothelial cells, in priming the metastatic potential of melanoma cancer cells [96].

Glutathione (GSH) is involved in cell protection against free radicals, and is particularly relevant in cancer cells by regulating tumorigenic mechanisms such as DNA synthesis, cell proliferation, drug resistance, and cytokine production, among others [97]. Importantly, IL-6 in the highly metastatic B16 melanoma F10 (B16-F10) cell line induces the production of GSH and its transport through the blood circulation to the brain metastatic growing foci, facilitating their growth in the brain [75]. The elevated expression of heparanase (HPSE) in melanoma cells has also been associated with increased cell growth, angiogenesis, and metastasis to the brain [98]. Interestingly, suppressing HPSE RNA expression has been shown to reduce melanoma cell migration, invasion, and adhesion capacities by inhibiting the expressions of IL-8 and CXCL1, as well as the activation of the MAPK signaling pathway [77]. Additional studies demonstrate that the stress hormone norepinephrine stimulates the growth and metastatic capacity of melanoma cells, in part by inducing the production of IL-6, IL-8, and VEGF [99]. Accordingly, IL-8 induced VEGFA angiogenic activity and increased the aggressiveness of malignant melanoma cells. Nonetheless, the growth and invasion of melanoma cells into the brain parenchyma relied primarily on the vascular co-option, controlled by the expression of the matrix metalloproteinases MMP-2 and MMP-9 [78]. Indeed, the brain metastatic melanoma-microglia interaction altered the secretion of vascularization-promoting factors including angiopoietin-2 or IL-8 from melanoma cells, and of GDF15 (growth/differentiation factor 15, also known as Macrophage inhibitory cytokine-1 or MIC-1) and other inflammation-related cytokines from microglia cells, favoring the metastatic process [100,101,102]. Previous works also indicated that metastatic melanoma cells secrete a large amount of TNF-α, IL-6, IL-12, IFN-γ, VEGF, eotaxin, and RANTES, triggering a cascade of effects that include the increase of MMP-2 enzymatic activity and tumor cell aggressiveness [76]. Similarly, IL-33 affects the progression of malignant melanoma cells by binding to its receptor ST2 and inducing tumor cell proliferation, migration, and invasion through MMP-2, MMP-9, and ERK1/2 phosphorylation [79]. IL-1β has also been shown to be upregulated in many solid tumors, including melanoma, and is associated with angiogenesis, invasiveness, and poor patient survival [103,104]. Mechanistically, this process is regulated by the IL-1β-producing myeloid cells, which subsequently activate endothelial cells to produce proangiogenic factors like VEGF, modulating the inflammatory brain microenvironment of the tumor and inducing an enhanced angiogenesis and tumor progression [80]. The efficacy of IFN-α2β and IFN-β1α in exerting an antitumor effect was shown against malignant human melanoma xenograft models. Indeed, IFN-β1α showed a strong anti-proliferative and pro-apoptotic effect, whereas IFN-α2β inhibited tumor growth metastases through the inhibition of lymphangiogenesis. Interestingly, both IFN-α2β and IFN-β1α decreased in-vitro and in-vivo VEGF-C and VEGF receptor-3 expression [81].

STAT3 activity is higher in human brain metastatic cells than in primary melanoma cells, and its activation induces angiogenesis, cell invasion, MMP-2 secretion, cytokine expression, and immune suppression, that contribute to their brain metastatic potential [105]. The inhibition of STAT3 signaling using the inhibitor WP1193 in brain metastatic melanoma patient samples induced the antitumor activity of IFN-α by enhancing both innate and adaptive cytotoxic T-cell activities in these cancer cells [82]. In melanoma cell lines, the loss of the suppressor of cytokine signaling-1 (SOCS-1) expression resulted in elevated STAT3 signaling and the overexpression of MMP-2, bFGF, and VEGF, leading to an enhanced invasion and angiogenesis of melanoma cells, and consequently promoting melanoma brain metastases [83]. The axis IL-17A-STAT3 also plays a role in the interaction between melanoma cells and microglia. Indeed, IL-17A promotes angiogenesis and induces IL-6 production in murine melanoma models, which in turn activates STAT3, upregulating the expression of angiogenesis and survival-supporting genes [69,106]. These results suggest that STAT3 activation may be, at least in part, responsible for melanoma brain metastasis occurrence that has been previously observed in a study of 216 autopsied metastatic melanoma specimens [107]. Importantly, brain-metastasizing melanoma cells can reprogram astrocytes to express the pro-inflammatory cytokine IL-23, which upregulates MMP-2 levels to facilitate melanoma cell migration and invasion into the brain parenchyma [84]. Thus, reduced expression levels of MMP-2 in melanoma cells resulted in the inhibition of IL-23-induced invasiveness.

TGF-β plays a complex role during tumorigenesis, either acting as a tumor suppressor through its broad anti-proliferative potential or as a tumor promoter either via direct effects on tumor cell aggressiveness or indirectly by modulating stromal responses, angiogenesis, and immune surveillance. In melanoma mouse models, an elevated TGF-β secretion was detected in tumor-associated microglia, inducing the tolerance of tumor cells against T cell cytotoxicity [85]. In addition, the expression of the TGF-β-receptor ligand TGF-β2 seems to play a critical role in melanoma brain metastases, as demonstrated in different mouse models [86]. Interestingly, TGF-β2 expression patterns were sufficient to spatially distinguish brain metastases arising from the B16 and K-1735 murine melanoma metastatic cell lines. B16 melanoma cells expressing low levels of TGF-β2 formed leptomeningeal diseases, whereas high K-1735 cells expressing high levels of endogenous TGF-β2 formed metastases in the brain parenchyma [86,108]. Of note, the modulation of TGF-β2 levels in both cell lines induced changes in their metastatic formation pattern, supporting the idea that TGF-β2 plays a pivotal role in the spatial distribution of melanoma metastases in the brain parenchyma.

Cytokines and chemokines can be used for the treatment of brain metastases. High-dose IL-2 is widely recognized in several studies to produce durable and favorable responses in metastatic melanoma, including patients with brain metastases [109]. Biochemotherapy with temozolomide, cisplatin, vinblastine, subcutaneous IL-2, and IFN-α in patients with brain metastatic melanoma was well tolerated but showed a modest antitumor activity [110]. Similarly, low-dose chemobiotherapy with temozolomide, GM-CSF, IFN-α2β, and recombinant IL-2 produced clinical responses in patients with metastatic melanoma and may protect against the development of brain metastases [111]. In another study, the sequential combination of fotemustine, cisplatin, IFN-α, and IL-2 showed acceptable clinical activity, especially in melanoma brain metastatic patients [112]. This was similar to the effects shown after the sequential combination of cisplatin, vinblastine, DTIC with IL-2, and IFN-α [113]. Additionally, patients with metastatic melanoma receiving high-dose IL-2 plus the gp100:209-217(210M) peptide vaccine had a higher response rate and longer progression-free survival than single regimen-treated patients [114]. Adoptive cell therapy with a nonmyeloablative preparative regimen using either tumor-infiltrating lymphocytes or T-cell receptor-transduced cells, combined with IL-2, can mediate a complete and durable regression of melanoma brain metastases in patients [115]. Other therapeutic regimens combining pegylated IFN-α-2α and dacarbazine [116], pegylated IFN-α-2β and temozolamide [117], and IFN-α-2β and tremelimumab [118] have been proven to be effective in advanced melanoma patients, with acceptable toxicity and promising durable antitumor activity. In another study, the intratumoral administration of human IL-12 encoded by a vector derived from the canarypox virus (ALVAC-IL-12) was well tolerated and resulted in a measurable biologic response in patients with brain metastatic melanoma [119]. Interestingly, the combined effects of IL-12 and EMD121974 (Cilengitide), a selective integrin αvβ3 antagonist, in melanoma cells significantly inhibited their brain metastatic capacity [120]. However, a prospective comparison of these therapeutic regimens is needed to confirm all these observations in patient samples.

## 5. Conclusions

**Figure 1:**
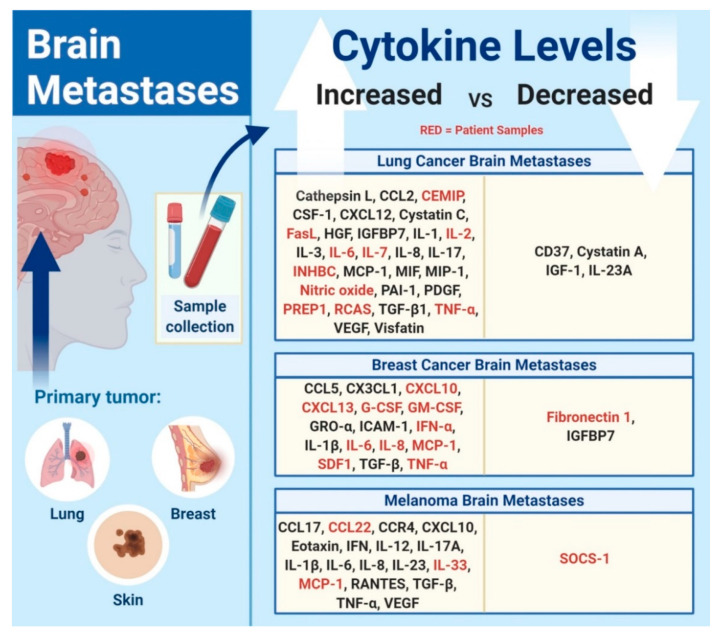
Cytokine and chemokine levels in brain metastases. Identifying the different levels of cytokines and chemokines in brain metastases from different primary tumors allows for the differentiation between patients with and without brain metastases. Red-colored cytokines correspond to studies that are based on patient samples.

Cytokines and chemokines are key multifunctional mediators that affect the immune-cell infiltration into brain metastases and impact the process of metastatic cancer cell survival in the brain (Figure 1). The dual functionality of chemokines in brain metastases may display both tumor-promoting and tumor-suppressive capabilities. Therefore, further exploration of the regulatory mechanisms that control the pro-tumor and anti-tumor activities of cytokines and chemokines in the setting of brain metastases is warranted. This will allow for the design of therapeutic molecules that can effectively target the tumorigenic pathways in patients with brain metastases.

Early diagnosis is crucial for the optimal treatment of patients with brain metastases. Employing identified cytokines and chemokines in biomarker panels can offer a rapid detection of brain metastases. Nevertheless, further studies and trials are needed to determine the relative accuracy of detection and to characterize the molecular foundation of circulating tumor cells. In addition, future works might explore how the levels of these cytokines in patients with brain metastases differ from patients with other inflammatory diseases (i.e., sepsis, rheumatoid arthritis, etc.). This will allow for the characterization of cytokine profiles that are specific for brain metastasis.

Despite some encouraging preclinical studies, chemokine-targeted therapy for the treatment of patients with brain metastases is still far from reach. Clinical trial of agents that target a single cytokine or cytokine receptor did not improve therapeutic outcomes in patients with other diseases, such as chronic inflammatory diseases or diabetes. The reason may be due to the fact that chemokines generally bind to multiple receptors. Inhibiting ligand activity may affect other essential cellular processes. In addition, it is important to consider the previous therapies that the patients received before the assessment of the cytokine/chemokine profile. In patients with cancer, chemotherapy and radiation therapy can affect the immune response and thus alter cytokine/chemokine production. As such, it is difficult to draw conclusions on the absolute effect of cytokines and chemokines when patients have undergone different cancer treatments. It would also be important to consider how soon after treatment the serum samples were collected for cytokine/chemokine profiling. Limiting the variabilities in patient data can allow us to draw solid conclusions in the future.

Using chemokine targeting agents with existing cancer therapies, including immunotherapies, might show synergistic therapeutic effects. Directly targeting both pro-tumor and antitumor chemokine–chemokine receptor signaling pathways in combination with other immunotherapies shows clinical benefits in patients with cancer [121,122,123,124]. Nevertheless, more preclinical studies and patient trials are required to bring this combination approach into clinical application in the setting of brain metastases.

## Figures and Tables

**Table 1:** Cytokines reported to have a role in lung cancer brain metastases.

Cytokine	Role	Model	Reference
TGF-β1	Promote EMT	In vitro	[11]
	Damage the endothelial glycocalyx, which subsequently improves the transmigration of metastasizing cells across the blood-brain barrier (BBB)	In vitro	[12]
SMAD6	GG genotype of SMAD6 rs12913975 and TT genotype of INHBC rs4760259 are associated with an increased risk of brain metastases	Patient samples	[13]
INHBC			
PREP1	EMT inducer and is a pro-metastatic transcription factor that acts by controlling the TGF-β-SMAD3 pathway	Patient samples	[14]
CCL2	Induces visfatin upregulation	In vitro	[15]
Visfatin	Mediates the transmigration of small-cell lung cancer (SCLC) cells across the BBB	In vitro	[15]
TNF-α	Enhances the adhesion of metastasizing lung cancer cells to the brain endothelial cells	Patient samples	[16]
Cystatin C	Damages the endothelial membrane and improves the transmigration of metastasizing cells across the BBB	In vitro	[12]
Cathepsin L			
IGFBP7	Improves the transmigration of metastasizing cells across the BBB	In vitro	[12]
VEGF			
CEMIP	Upregulates pro-inflammatory cytokines to promote brain vascular remodeling	Patient samples	[17]
MIF	Activate astrocytes in the tumor microenvironment and increases the expression of IL-6 receptors	In vitro	[18]
IL-8			
PAI-1			
IL-6	Promote tumor cell proliferation through the STAT3 pathway	In vitro	[18]
	Induces PD-L1 expression in myeloid cells	Patient samples	[19]
CSF-1	Reprograms myeloid cells, specifically, into tumor-promoting macrophages in the brain parenchyma	In vitro	[20]
IL-2	Regulate the IFN-γ responses to the tumor surface antigen mesothelin	Patient samples	[21]
IL-7			
Nitric oxide	Remodel the cytoskeleton and promote the mobility of lung cancer cells	Patient samples	[22]
RCAS	Induce apoptosis of NK/T cells and promote immune evasion	Patient samples	[23]
FasL			
HGF	Enhance tumorigenicity and direct metastases to the brain	In vitro	[24]

**Table 2:** Cytokines reported to have a role in breast cancer brain metastases.

Cytokine	Role	Model	Reference
CXCL13	Increases the permeability of metastasizing breast cancer cells across the blood-brain barrier (BBB)	Patient samples	[35]
CCL4	Facilitate the transmigration of breast cancer cells across the BBB	In vitro	[36]
CCL5		In vitro	[36]
		In vivo (mouse)	[37]
ICAM-1		In vivo (mouse)	[38]
IL-6 IL-8 CCL2		Patient samples	[39]
GRO-α		In vivo (mouse)	[40]
G-CSF	Recruits Arg1+ and PD-L1+ immunosuppressive neutrophils into the brain to drive metastasis outgrowth	Patient samples	[41]
VEGF	Drives angiogenesis and growth of brain metastases	In vivo (mouse)	[42]
SDF1	Acts on microglia to support the invasion of breast cancer cells into the brain	Patient samples	[43]
	Upregulates VEGF, MMP9, SLUG, E-cadherin, ATG5, LC3-II and p62/SQSTM1 to promote tumor cell adaptation and progression in the brain	Patient samples	[44]
		In vitro	[45,46,47,48]
MCP-1	Promotes migration and infiltration of macrophage into the brain through its receptor CCR2	Patient samples	[49,50]
GM-CSF	Facilitate the transmigration of breast cancer cells across the BBB	Patient samples	[51]
	Enhances microglial proliferation in the tumor microenvironment	In vivo (rat)	[52]
CX3CL1	Attracts macrophages and microglial cells into the tumor microenvironment	In vivo (mouse)	[53]
	Stimulate brain microvessel endothelial cells, leading to increased permeability of the BBB	In vitro	[54,55]
IFNα	Activate the STAT1 and NF-κB pathways in brain metastatic cells, thereby promoting tumor growth and resistance	Patient samples	[56]
TNF			
TGF-β1	Regulates breast cancer cell invasion and colonization in the brain	In vitro	[57]
Fibronectin 1	Involved in tumor progression and invasion	Patient samples	[58]
IGFBP7	Suppressed in breast cancer brain metastatic cells in the brain due to its tumor suppressor properties	In vitro	[59]
CXCL10	Mediates recruitment of immune-suppressive CNS-myeloids to brain metastases	Patient samples	[60]

**Table 3:** Cytokines reported to have a role in melanoma brain metastases.

Cytokine	Role	Model	Reference
IL-17A	Promotes angiogenesis and induces IL-6 production	In vitro	[69]
CXCL10	Modulates the migration of monocytes, macrophages, T cells, and NK cells to the brain	In vivo (mouse)	[70]
CCL17	Increases tumorigenicity and micrometastasis formation in the brain	In vivo (mouse)	[71]
CCL2	Recruits cytotoxic T lymphocytes to the metastatic melanoma site and induces an immune-mediated protective role	In vitro	[72]
	Recruits myeloid cells that prime the growth of metastatic melanoma cells in the brain	Patient samples	[73]
CCL22	Regulates the AKT phosphorylation pattern and subsequent tumor cell survival and proliferation	Patient samples	[74]
IL-6	Induces the production of GSH in melanoma cells, facilitating their growth in the brain	In vivo (mouse)	[75]
	Triggers MMP-2 enzymatic activity in the tumor microenvironment	In vitro	[76]
IL-8	Increases melanoma cell migration, invasion, and adhesion capacities, and activates MAPK signaling pathway	In vivo (mouse)	[77]
	Induces VEGFA-mediated angiogenesis and vascular co-option controlled by MMP-2 and MMP-9	In vivo (mouse)	[78]
TNF-α IFN-γ	Enhances the invasion of metastatic melanoma cells and increases tumor cell aggressiveness	In vitro	[76]
VEGF Eotaxin RANTES IL-12	Trigger MMP-2 enzymatic activity that enhances the invasion of metastatic melanoma cells and increases tumor cell colonization	In vitro	[76]
IL-33	Binds to ST2 receptor and induces melanoma proliferation, migration, and invasion through MMP-2, MMP-9, and ERK1/2 phosphorylation	Patient samples	[79]
IL-1β	Induces VEGF production by endothelial cells, modulating the inflammatory brain microenvironment of the tumor and enhancing angiogenesis and tumor progression	In vivo (mouse)	[80]
IFN-α2β	Inhibit lymphangiogenesis-mediated melanoma metastasis by decreasing VEGF-C and VEGF receptor-3 expression	In vivo (mouse)	[81]
IFN-β1α			
IFN-α	Enhances both innate and adaptive cytotoxic T-cell activities	In vivo (mouse)	[82]
SOCS-1	Inhibits Stat3 signaling and downregulates MMP-2, bFGF, and VEGF, leading to decreased invasion and angiogenesis	Patient samples	[83]
IL-23	Upregulates MMP-2 to facilitate melanoma cell migration and invasion into the brain parenchyma	In vivo (mouse)	[84]
TGF-β	Induces tolerance of melanoma cells against T cell cytotoxicity	In vitro (mouse)	[85]
	Plays a pivotal role in the spatial distribution of melanoma cells in the brain parenchyma	In vivo (mouse)	[86]
